# Insulin activates hepatic Wnt/β-catenin signaling through stearoyl-CoA desaturase 1 and Porcupine

**DOI:** 10.1038/s41598-020-61869-4

**Published:** 2020-03-20

**Authors:** Régine Cabrae, Céline Dubuquoy, Michèle Caüzac, Lucille Morzyglod, Sandra Guilmeau, Bénédicte Noblet, Bruno Fève, Catherine Postic, Anne-Françoise Burnol, Marthe Moldes

**Affiliations:** 1 Inserm, U1016, Institut Cochin, CNRS UMR 8104,75014, Université Paris Descartes, Sorbonne Paris Cité, 75006 Paris, France; 2 Sorbonne Université, Inserm, Institute of Cardiometabolism and Nutrition, ICAN, Centre de Recherche Saint-Antoine, CRSA, F-75012 Paris, France; 3 Sorbonne Université, Inserm, Centre de Recherche Saint-Antoine (CRSA), Institute of Cardiometabolism and Nutrition (ICAN), AP-HP, Saint-Antoine Hospital, Department of Endocrinology, Paris, France

**Keywords:** Insulin signalling, Homeostasis

## Abstract

The Wnt/β-catenin pathway plays a pivotal role in liver structural and metabolic homeostasis. Wnt activity is tightly regulated by the acyltransferase Porcupine through the addition of palmitoleate. Interestingly palmitoleate can be endogenously produced by the stearoyl-CoA desaturase 1 (SCD1), a lipogenic enzyme transcriptionally regulated by insulin. This study aimed to determine whether nutritional conditions, and insulin, regulate Wnt pathway activity in liver. An adenoviral TRE-Luciferase reporter was used as a readout of Wnt/β-catenin pathway activity, *in vivo* in mouse liver and *in vitro* in primary hepatocytes. Refeeding enhanced TRE-Luciferase activity and expression of Wnt target genes in mice liver, revealing a nutritional regulation of the Wnt/β-catenin pathway. This effect was inhibited in liver specific insulin receptor KO (iLIRKO) mice and upon wortmannin or rapamycin treatment. Overexpression or inhibition of SCD1 expression regulated Wnt/β-catenin activity in primary hepatocytes. Similarly, palmitoleate added exogenously or produced by SCD1-mediated desaturation of palmitate, induced Wnt signaling activity. Interestingly, this effect was abolished in the absence of Porcupine, suggesting that both SCD1 and Porcupine are key mediators of insulin-induced Wnt/β-catenin activity in hepatocytes. Altogether, our findings suggest that insulin and lipogenesis act as potential novel physiological inducers of hepatic Wnt/β-catenin pathway.

## Introduction

Liver plays a central role in the control of energy homeostasis as it contributes to the maintenance of glycemia upon varying nutritional conditions through the regulation of glucose and lipid metabolism^1,2^. During fasting, hepatic glucose production ensures a constant plasma glucose concentration and energy supply to peripheral tissues, whereas in the post-prandial period increased plasma insulin concentration favors liver glucose uptake restoring glycogen contents and stimulating lipid synthesis through *de novo* lipogenesis and fatty acid esterification^2^. These opposite metabolic functions are finely regulated by hormonal, nutrient and molecular gradients existing along the liver acini^3,4^. Among these molecular gradients, the Wnt/β-catenin pathway participates to liver functional zonation, and to hepatic regeneration and proliferation^5–7^. Interestingly, this signaling pathway is also involved in hepatic metabolism since mutations in TCF7L2 or LRP6 genes, encoding a nuclear partner of β-catenin and a co-receptor of Wnt, respectively, were associated with an increased risk to develop diabetes and hyperlipidemia^8–10^.

The Wnt/β-catenin signaling cascade is initiated by Wnt morphogens binding to Frizzled (Fzd) receptors, which leads to β-catenin stabilization and translocation into the nucleus. In association with its nuclear partner TCF/LEF (*T-cell factor/lymphoid enhancing factor*), β-catenin trans-activates Wnt target genes through TCF-responsive elements (TRE)^6^. Secretion of Wnt ligands and receptor binding are limiting steps in Wnt signaling activity and are tightly regulated by series of Wnt post-translational modifications, such as acylation^11,12^. Addition of the fatty acid palmitoleate on a serine residue, which is conserved among the Wnt ligands, is an essential event in their intracellular trafficking from endoplasmic reticulum (ER) to Golgi and their consecutive secretion^13^. This reaction is catalyzed by the acyltransferase Porcupine, a member of the MBOAT (*Membrane‐Bound‐O‐Acyl‐Transferase*) family of proteins^14–16^. Palmitoleate is the product of the desaturation of palmitate by the stearoyl-CoA-desaturase 1 (SCD1), while palmitate is synthetized by fatty acid synthase (FAS)^17^. Both enzymes were shown to participate to Wnt biogenesis^18,19^. Interestingly, FAS and SCD1 are enzymes of the *de novo* lipogenesis pathway, they are mainly up-regulated upon refeeding and more particularly by insulin and glucose^20^.

The aim of the current study was to investigate the effect of insulin on the Wnt/β-catenin signaling pathway in liver and hepatocytes in culture, a topic poorly documented so far in this major insulin target organ. We studied the regulation of hepatic Wnt/β-catenin pathway activity by nutritional conditions and demonstrated that under physiological conditions insulin induces the Wnt pathway by stimulating the PI3K/mTORC1 (*target of rapamycin complex 1)* signaling pathway and lipogenesis. Activated by insulin, the lipogenic enzyme SCD1 acts as a palmitoleate supplier for Porcupine, which then acylates Wnt ligand in hepatocytes. Altogether, our findings unravel the insulin-dependent *de novo* lipogenesis as a novel physiological inducer of the hepatic Wnt/β-catenin pathway.

## Results

### High carbohydrate refeeding activates the Wnt/β-catenin pathway in mouse liver

**Figure 1:**
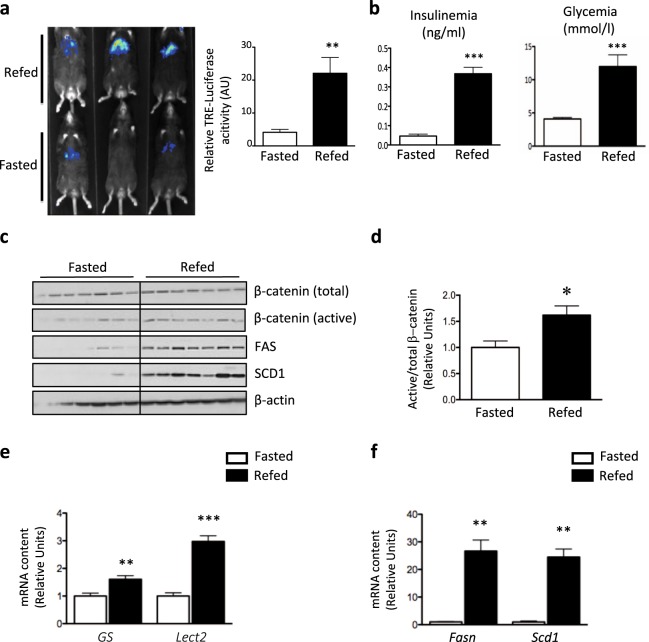
Wnt/β-catenin pathway activity is activated by refeeding in mouse liver. (**a**) Mice infected with Adv-TRE-Luc and Adv-RSV-β-gal were studied 4 days later in fasted or refed state as described in “Methods”. *In vivo* imaging of hepatic TRE-Luciferase activity (left panel) and quantification of luciferase activity (right panel) were performed. Results are expressed as percent of the ratio firefly luciferase/β-galactosidase. (**b**) Plasma insulin and blood glucose concentrations were measured in fasted and refed conditions. (**c**,**d**) Western blot analysis of liver protein expression in lysates from fasted and refed mice (**c**) and quantification of active-to-total β-catenin in liver of fasted and refed mice. (**d**) β-Actin antibody was used as a loading control (n = 7/group). (**e,f**) RT-qPCR analysis of Wnt target genes (*GS* and *Lect2)* (**e**) and of lipogenic gene (*Fasn* and *Scd1*) (**f**) expression in liver of fasted and refed mice. Results are the mean ± SEM (n = 5–7/group). **p < 0.01, ***p < 0.001 for refed *vs* fasted mice.

The activity of the Wnt/β-catenin pathway was monitored in mouse liver by *in vivo* imaging using an adenovirus containing TCF-responsive elements upstream a minimal promoter and a luciferase reporter gene (Adv-TRE-Luc). Fasted mice displayed low TRE-Luc activity, whereas upon refeeding with a high carbohydrate diet, leading to elevated glucose and insulin plasma concentrations, luciferase activity was induced by 5.5-fold (Fig. 1a,b). Accordingly, the protein content of the active form of β-catenin was enhanced during the nutritional challenge (Fig. 1c and quantification on Fig. 1d**)**, although β-catenin mRNA or total protein levels remained unchanged (Supplementary Fig. 1). As a consequence, the expression of Wnt target genes *GS* (*Glutamine Synthetase*) and *Lect2* (*leukocyte cell-derived chemotaxin 2*) was induced, as shown by RT-qPCR (Fig. 1e). Notably, upon refeeding the enhanced Wnt/β-catenin activity was concomitant with the sharp induction of lipogenic gene expression, *Fasn* (*fatty acid synthase*) and *Scd1* (*stearoyl-CoA desaturase*) (Fig. 1c,f). Altogether, these results suggest that refeeding conditions activates Wnt/β-catenin pathway in mice liver.

### Hepatic Wnt/β-catenin pathway is activated by insulin

**Figure 2:**
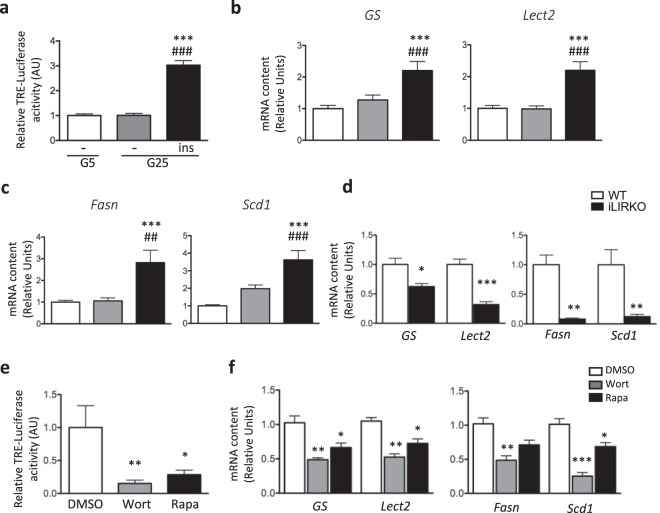
Insulin stimulates hepatic Wnt signaling activity *in vitro* and *in vivo*. (**a**) Primary mouse hepatocytes infected with Adv-TRE-Luc and Adv-RSV-β-gal were cultured with either 5 mM (G5) or 25 mM glucose (G25) in the presence or the absence of insulin (ins, 100 nM) for 24 hours. Quantification of luciferase activity is expressed as percent of the ratio firefly luciferase/β-galactosidase. (**b**,**c**) RT-qPCR analysis of Wnt target genes (*GS* and *Lect2*) (**b**) and lipogenic genes (*Fasn* and *Scd1*) (**c**) expression in primary mouse hepatocytes incubated in 5 mM or 25 mM glucose in the presence or the absence of insulin. Results for a-c are the mean ± SEM (n = 3–5). ***p < 0.001 *vs* G5; ^##^p < 0.01, ^###^p < 0.001 *vs* G25. (**d**) RT-qPCR analysis of Wnt target genes (*GS* and *Lect2*, left panel) and lipogenic genes (*Fasn* and *Scd1*, right panel) expression in liver from iLIRKO and control mice. Results are the means ± SEM (n = 9–10/group). *p < 0.05, ***p < 0.001, iLIRKO *vs* control mice. (**e,f**) Mice infected with Adv-TRE-Luc and Adv-Rsv-β-gal were treated with either DMSO, wortmannin (Wort) or rapamycin (Rapa) and sacrificed in the refed state. (**e**) Quantification of TRE-Luciferase activity after *in vivo* imaging of infected mice. (**f**) RT-qPCR analysis of Wnt target genes (*GS* and *Lect2*) (left panel) and lipogenic genes (*Fasn* and *Scd1*)(right panel) expression in liver from treated mice. Results are the mean ± SEM (n = 8–9/group). *p < 0.05, **p < 0.01, ***p < 0.001 for inhibitor-treated mice compared to DMSO control mice.

To investigate the contribution of insulin and/or glucose on Wnt signaling activity, mouse primary cultured hepatocytes were infected with the Adv-TRE-Luc construct and incubated in low (5 mM) or in high (25 mM) glucose concentration either in the presence or the absence of insulin (100 nM). Of note, the viability of hepatocytes was not altered in the absence of insulin during culture time period (Supplementary Fig. 2). As shown in Fig. 2a, TRE-Luc activity was not modified by high glucose concentration, while insulin had a significant stimulatory effect. The expression of Wnt target genes *Lect2* and *GS* was also enhanced in the presence of insulin and 25 mM glucose, similarly to lipogenic genes *Fasn* and *Scd1* (Fig. 2b,c), suggesting that high concentrations of insulin and glucose can stimulate the Wnt/β-catenin pathway in hepatocytes. The contribution of insulin to the activation of liver Wnt/β-catenin pathway was further established *in vivo* using a mouse model of inducible liver specific insulin receptor knock-out (iLIRKO)^21^ (Supplementary Fig. 3a). Two weeks after tamoxifen injection, mice displayed increased insulinemia, while glycemia was not significantly altered (Supplementary Fig. 3b). As expected, *Fas* and *Scd1* mRNA and protein concentrations were dramatically reduced in refed iLIRKO mice compared to their control littermate, illustrating the drastic inhibition of liver insulin signaling in these mice (Fig. 2d right panel and Supplementary Fig. 3a). Importantly, *GS* and *Lect2* expression was also significantly reduced in liver of iLIRKO mice (Fig. 2d left panel).

Liver insulin actions on metabolic pathway are mainly mediated by the activation of the PI3K-Akt-mTORC1 signaling cascade^22,23^. To investigate the role of this pathway in Wnt/β-catenin activation, we used wortmannin, Akti or rapamycin to inhibit PI3K, Akt, and mTORC1, respectively. The efficiency of these inhibitors was validated on insulin signaling in mouse primary hepatocytes (Supplementary Fig. 3c). Interestingly, pre-incubation of cultured hepatocytes with these inhibitors blocked the induction by insulin of TRE-Luc activity (Supplementary Fig. 3d). To further investigate *in vivo* the role of PI3K-mTORC1 signaling on Wnt/β-catenin activation, mice infected with Adv-TRE-Luc were treated with wortmannin or rapamycin. As shown in Fig. 2f (right panel) liver *Fasn* and *Scd1* gene expression was significantly decreased upon treatment with the PI3K-mTORC1 inhibitors. Under these conditions, TRE-Luc activity was strikingly inhibited and expression of Wnt target genes *GS* and *Lect2* was decreased by 30–50% in mice liver (Fig. 2e,f left panel). Altogether, these data reveal that the insulin signaling pathway regulates hepatic Wnt/β-catenin activity. Notably, they also suggest an interesting correlation between Wnt canonical pathway activity and induction of the lipogenic transcriptional program upon insulin challenge.

### Palmitoleate production through SCD1 is implicated in insulin-induced hepatic Wnt/β-catenin pathway activity

**Figure 3:**
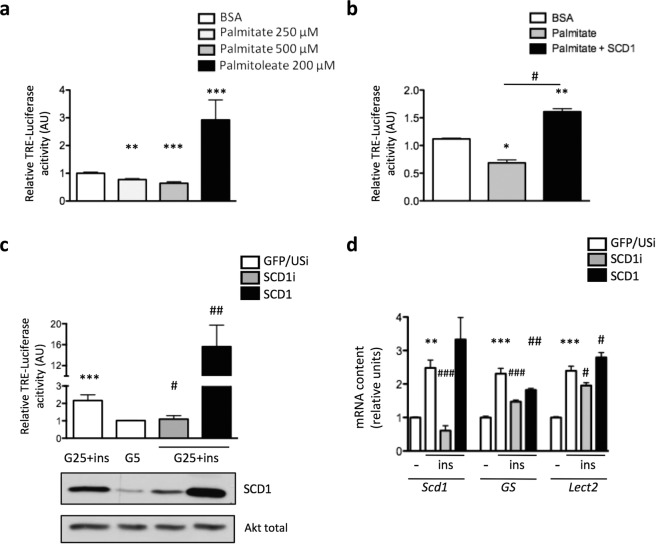
SCD1-mediated palmitoleate production enhances insulin-induced Wnt pathway activity in hepatocytes. Primary mouse hepatocytes were infected with Adv-TRE-Luc and Adv-RSV-β-gal and incubated in different conditions as indicated. Luciferase assays were performed and results are expressed as percent of the ratio firefly luciferase/β-galactosidase. (**a**) Adv-TRE-Luc-infected primary hepatocytes were incubated with palmitate, palmitoleate or control 0.6% BSA for 24 h as indicated. **p < 0.01, ***p < 0.001 *vs* BSA. (**b**) TRE-Luc-expressing primary hepatocytes were infected with either SCD1 or control GFP adenoviruses and incubated in the presence of palmitate (250 μM) or BSA for 24 h. Results are the mean ± SEM (n = 3). *p < 0.05, **p < 0.01 *vs* BSA; ^#^p < 0.05 *vs* palmitate. (**c**) TRE-Luc-expressing primary hepatocytes were infected with GFP/USi, SCD1i or SCD1 adenoviruses and incubated in 25 mM glucose (G25) in the presence of the absence of insulin (ins) for 24 h. Results are the mean ± SEM (n = 3). ***p < 0.001 *vs* G25; ^#^p < 0.05, ^###^p < 0.001 *vs* G25 + ins. The Western blot shows SCD1 protein content in hepatocyte lysates from the different conditions. β-Actin antibody was used as a loading control. (**d**) RT-qPCR analysis of *Scd1* and of Wnt target genes (*GS* and *Lect2*) expression in primary hepatocytes infected with the indicated adenoviruses and incubated in 25 mM glucose in the presence of the absence of insulin (ins) for 24 h. Results are the mean ± SEM (n = 3). **p < 0.01, ***p < 0.001 *vs* G25; ^#^p < 0.05, ^##^p < 0.01, ^###^p < 0.001 *vs* G25 + ins.

Wnt secretion and activity are tightly regulated by palmitoylation and SCD1^12,18^. Palmitoleate is produced by the lipogenic enzyme SCD1 through palmitate desaturation^24^. In the hepatic context, we investigated whether palmitoleate could be a mediator connecting insulin-induced hepatic lipogenesis to enhance Wnt canonical pathway. Primary hepatocytes infected with Adv-TRE-Luc were incubated in the presence of either palmitoleate (200 μM) or palmitate (250 or 500 μM). The addition of palmitoleate significantly stimulated Wnt/β-catenin activity by 3-fold, whereas palmitate decreased TRE-Luc activity (Fig. 3a). Importantly, when SCD1 was overexpressed, the addition of palmitate to the media enhanced Wnt/β-catenin activity (Fig. 3b). These data suggest that palmitoleate, either added exogenously or produced by SCD1-mediated desaturation of palmitate, stimulates Wnt/β-catenin pathway activity in mouse hepatocytes. The contribution of SCD1 to Wnt signaling activation was then investigated using an adenoviral strategy to downregulate or increase SCD1 expression. SCD1 content was validated by Western blot and RT-qPCR analysis (Fig. 3c lower panel and Fig. 3d). SCD1 silencing abolished the insulin-mediated activation of Wnt signaling, while SCD1 overexpression enhanced the effect of insulin on TRE-Luc activity (Fig. 3c upper panel). Accordingly, expression of the Wnt target genes *GS* and *Lect2* was repressed after SCD1 knockdown and *Lect2* was induced upon SCD1 overexpression (Fig. 3d). Taken together these results suggest that in hepatocytes, insulin-induced Wnt pathway activity may require SCD1 activity and the associated palmitoleate production.

### Porcupine contributes to the insulin-induced Wnt/β-catenin pathway in hepatocytes

**Figure 4:**
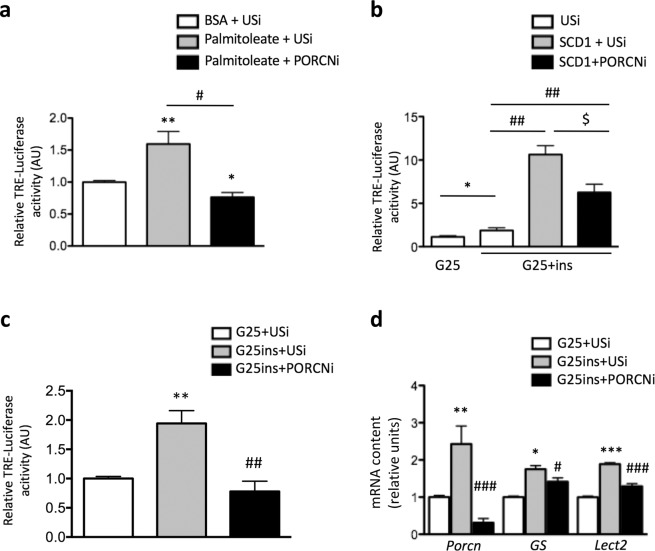
Porcupine is implicated in insulin-induced hepatic Wnt/β-catenin pathway activation. (**a**) TRE-Luc-expressing primary hepatocytes were infected with PORCNi or USi adenoviruses and incubated with either BSA (0.6%), palmitoleate (200 μM) for 24 h. Luciferase assays were performed and results are expressed as percent of the ratio firefly luciferase/β-galactosidase. Results are the mean ± SEM (n = 3). *p < 0.05, **p < 0.01 *vs* BSA; ^#^p < 0.05 *vs* palmitoleate + USi. (**b**) TRE-Luc-expressing primary hepatocytes were infected with USi + GFP, SCD1 + USi or SCD1 + PORCNi adenoviruses and incubated with G25 or G25 plus insulin for 24 h. Luciferase assays were performed and results are expressed as percent of the ratio of firefly luciferase/β-galactosidase (n = 3). *p < 0.05 *vs* G25; ^##^p < 0.01 *vs* G25 + ins and ^$^p < 0.05 *vs* SCD1 + USi. (**c**,**d**) TRE-Luc-expressing primary hepatocytes were infected with PORCNi or USi and incubated in G25 or G25 + ins for 24 h. (**c**) Luciferase assays and (**d**) RT-qPCR analysis of *PORCN*, *GS* and *Le****c****t2* gene expression were performed. Results in c and d are the mean ± SEM (n = 3–4). *p < 0.05, **p < 0.01, ***p < 0.001 *vs* G25; ^#^p < 0.05, ^##^p < 0.01, ^###^p < 0.001 *vs* G25 + ins.

The acyltransferase Porcupine (PORCN) was reported to catalyze Wnt palmitoylation in mouse fibroblasts or chick neural tube^12,14,25,26^. We thus investigated the role of Porcupine on the induction of Wnt signaling in insulin-sensitive primary mouse hepatocytes. Porcupine was efficiently knocked-down by an adenoviral shRNA targeting *Porcn* (PORCNi) (Fig. 4d and Supplementary Fig. 4a). Inhibition of *Porcn* expression abolished the stimulatory effect of palmitoleate on TRE-Luc activity (Fig. 4a), and decreased by 50% SCD1-induced Wnt/β-catenin activity in hepatocytes cultured in the presence of high insulin and glucose concentrations (Fig. 4b). Furthermore, insulin-induced TRE-Luc activity and expression of *GS* and *Lect2* were significantly reduced upon downregulation of PORCN in lipogenic conditions (Fig. 4c,d). Similar results were observed with the pharmacological inhibitor of PORCN, IWP1 (inhibitor of Wnt processing and secretion) (Supplementary Fig. 4b), confirming the role of Porcupine in insulin-induced Wnt/β-catenin activation. Of note, *Porcn* expression was enhanced in the presence of high concentrations of insulin and glucose (Fig. 4d). Altogether, our data provide evidence that in mouse hepatocytes insulin can activate Wnt/β-catenin pathway through the induction of palmitoleate synthesis and Porcupine.

## Discussion

**Figure 5:**
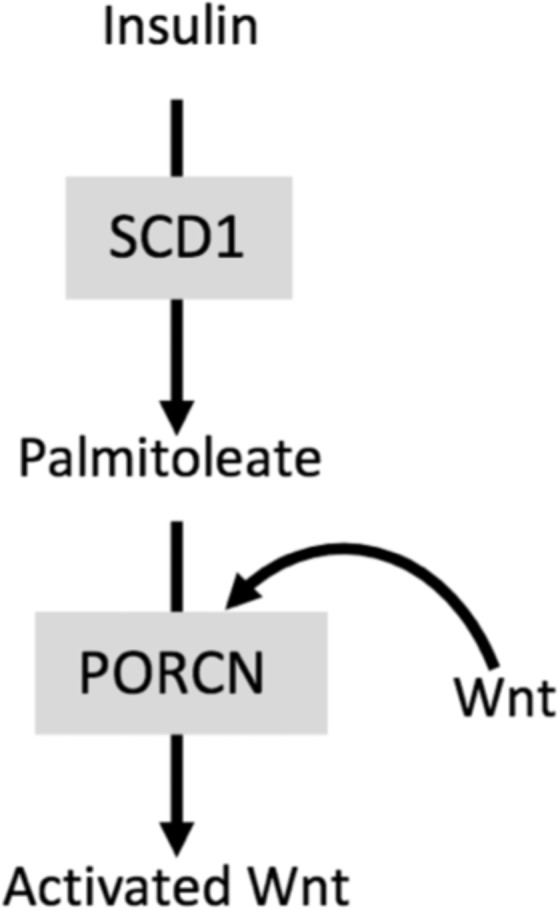
Model for the molecular mechanism involving insulin-induced hepatic Wnt signaling activity through SCD1, palmitoleate and Porcupine. Insulin stimulates *de novo* lipogenesis through PI3K/mTORC1 signaling, leading to the expression of the fatty acid desaturase SCD1. This lipogenic enzyme acts as a supplier of palmitoleate used as a substrate by the acyltransferase Porcupine to activate the Wnt signaling pathway. Thus, during refeeding, insulin is an upstream regulator of SCD1, which in concert with Porcupine may stimulate hepatic Wnt/β-catenin activity.

In the present study, we revealed a novel mechanism for the regulation of the Wnt/β-catenin pathway in hepatocytes, involving insulin signaling and lipid metabolism. We showed that the activity of the Wnt canonical pathway is controlled by nutritional conditions, and more particularly by insulin, as schematized in Fig. 5. Insulin stimulates *de novo* lipogenesis through PI3K/mTORC1 signaling, leading to the expression of the fatty acid desaturase SCD1 which acts as a supplier of palmitoleate and the acyltransferase Porcupine to activate the Wnt signaling pathway. These data suggest that during refeeding, insulin is an upstream regulator of SCD1, which in concert with Porcupine may stimulate hepatic Wnt/β-catenin activity.

In a physiological context, the regulation by insulin of the Wnt/β-catenin pathway may be linked to its crucial role in hepatic structural and metabolic homeostasis. Liver is a complex organ structurally organized in functional units called acini or hepatic lobules. Acini are differentially supplied with hormones and nutrients through the blood flow along the porto-central axis of the liver lobule. As a consequence, metabolic functions are spatially compartmentalized in liver acini with periportal (PP) hepatocytes involved in gluconeogenesis whereas perivenous (PV) hepatocytes favor glucose uptake and lipogenesis^3,4^. This metabolic zonation is also under the control of the Wnt/β-catenin pathway, which is activated in PV hepatocytes and blocked in PP hepatocytes by its inhibitor Apc (Adenomatous polyposis coli)^5^. Hepatocyte-specific β-catenin transgenic and knockout mice models revealed that Wnt/β-catenin dependent metabolic zonation is critical for liver response to metabolic stress such as starvation or chronic excess of energy intake^27^. Altogether, this suggests that Wnt signaling activation by insulin-promoted *de novo* lipogenesis can contribute to hepatic metabolic flexibility and favor integrated metabolic responses in liver according to nutritional changes.

Cross-talks between intermediates of the insulin signaling and Wnt/β-catenin pathway have previously been pointed out. In response to insulin stimulation, the adaptors IRS1/2 interact with Dishevelled2 (Dvl2), favoring Dvl2 stabilization and promoting canonical Wnt signaling^28^. In addition, insulin stimulates GSK3β (a common effector of both insulin and Wnt/β-catenin pathways) phosphorylation and inactivation through Akt, thereby altering its ability to inhibit glycogen metabolism and to promote β-catenin protein degradation^29–31^. Insulin signaling can thus control Wnt/β-catenin pathway activity through various step points, including the activation of signaling effectors, palmitoleate production through activation of lipogenesis, and Porcupine expression and/or activity.

In the present paper, we showed that palmitoleate is sufficient to trigger Wnt signaling activity in primary cultured hepatocytes. The effect of palmitoleate can be reproduced by the addition of palmitate in the presence of SCD1 or can be inhibited by the downregulation of the acyl-transferase Porcupine, suggesting that it is indeed the presence of palmitoleate, as a precursor of acylation, that is required for triggering Wnt signaling. Post-translational modification of Wnt is known to regulate β-catenin activity, and more particularly it is now well established that Wnt acylation, through the addition of a palmitoleate residue by Porcupine, is required for Wnt processing and action^12,32^. Palmitoleate has been described to act as an insulin sensitizing lipokine^33,34^, and amount of circulating ‘free’ palmitoleate in plasma has been positively associated with insulin sensitivity in humans. Furthermore, induction of SCD1 and production of palmitoleate can rescue insulin sensitivity in murine models of obesity and insulin resistance^35^. However, it has also been documented that excess lipogenesis is associated with fatty liver and insulin resistance, and that decreasing the lipogenic pathway through SCD1 inhibition can restore insulin sensitivity^36–38^. The role of palmitoleate, as a potentially metabolically toxic lipid or as a protective lipokine, may depend on the tissue where it is produced and on the physiological or pathophysiological context. Further studies are needed to clarify its role.

Metabolic diseases are characterized by a chronic insulin-resistant state, leading to hyperinsulinemia and potentially to dysglycemia. Interestingly, liver exhibits a “selective insulin resistance” characterized by a defect in the suppression of gluconeogenesis while insulin activation of lipogenesis is preserved. Kubota *et al*. proposed that differential distribution of insulin-receptor substrate proteins, IRS-1 and IRS-2 along liver acini contributes to this selective insulin resistance^39^. In the liver of mice submitted to a high-fat diet, insulin signaling is impaired in the PP zone, due to decreased IRS-2 expression. In the PV area, Wnt signaling stimulates the expression of its target gene IRS-1 therefore contributing to an active insulin signaling and maintaining *de novo* lipogenesis^39^. The activation of insulin and Wnt signaling pathways may finally worsen liver steatosis in a feed-forward loop in the pathological context of obesity and type 2 diabetes. Epidemiological studies suggest that obesity and diabetes are risk factors for hepatocellular carcinomas (HCC) development^40,41^. In this context, hyperinsulinemia could lead to the over-activation of the Wnt/β-catenin pathway, through the combined activity of SCD1 and Porcupine, and hence participate to tumor development. This is supported by the recent study of Lai *et al*. which described in hepatic stellate cells and hepatocellular carcinoma cells a positive-feedback loop where SCD enhances Wnt signaling through Lrp5 and Lrp6 stabilization and is itself regulated at the transcriptional level by Wnt signaling, contributing to hepatic fibrosis and tumor growth^42^. In addition, SCD1 expression is positively associated with the prevalence of prostate, lung and breast cancers^43–45^, and its inhibition decreases tumor cell proliferation *in vitro*^46–48^. However, in a therapeutical context, inhibition of SCD1 will lead to an increase in saturated fatty acids intracellular concentration, favoring lipotoxicity^49,50^. Porcupine seems thus a more promising therapeutic target to block the proliferative effects of Wnt^51^, encouraging the development of pharmacological selective modulators to target and handle certain cancers without altering normal cells^52–54^.

We showed that insulin could regulate the Wnt signaling activity in hepatocytes. This action involves the induction of the SCD1 enzyme as a provider of palmitoleate and the acyltransferase Porcupine to acylate the Wnt ligand. These findings establish insulin and the downstream lipogenic pathway as potential new physiological inducers of hepatic Wnt/β-catenin pathway. Moreover, the numerous points of inter-connection observed between the insulin and Wnt signaling pathways suggest that a tight regulation of these two pathways is required to maintain hepatic metabolic and structural homeostasis.

## Methods

All methods were carried out in accordance with relevant guidelines and regulations.

### Animals

Eight- to ten-week-old male C57BL/6 J mice purchased from Harlan Laboratories (Indianapolis, IN, USA) were adapted to the environment for one week before the study. The inducible liver IR knockout (iLIRKO) mice were described previously^21^. Fifteen-week-old male mice were studied 2 weeks after 3 consecutive injections with tamoxifen (1.5 mg/mouse). All mice were housed in colony cages with a 12 h/12 h light/dark cycle in a temperature-controlled environment (lights off at 3:00 pm). Mice had free access to water and regular diet (65% carbohydrate, 11% fat and 24% protein). For “fasting” and “refed” conditions, mice were either fasted for 24 h, or refed overnight on a regular diet with 20% glucose in tap water after 24 h fasting. For insulin signaling inhibitors experiments, mice were injected twice (18 hours and 2 hours) before sacrifice with wortmannin (2 mg/kg, InVivoGen, CA, USA) or rapamycin (4.5 mg/kg, InVivoGen). Livers were frozen in liquid nitrogen and kept at −80 °C until use. Plasma samples were frozen and stored at −80 °C until use. All procedures were carried out according to the French guidelines for the care and use of experimental animals. All animal studies were approved by the “Direction départementale des services vétérinaires de Paris”.

### Generation of the adenovirus

The TRE-Luc adenoviral vector was generated from the Super8xTOP-Flash plasmid containing 8 TCF-responsive elements (M50 Super 8x TOPFlash was a gift from Randall Moon (Addgene plasmid # 12456; Addgene, MA, USA)^55^, and subcloned into the Shuttle adenoviral vector (pAd-easy)^56^. Recombinant TRE-Luc, RSV-β-gal and GFP adenoviruses were produced by the Laboratoire de Thérapie Génique (Nantes, France). Recombinant adenoviral constructs sh-scramble (USi) and sh-PORCN (PORCNi) were purchased from VectorBiolabs (PA, USA) and produced by the Laboratoire de Thérapie Génique, respectively. SCD1 and shSCD1 (SCD1i) adenoviruses were purchased at GeneCust (Dudelange, Luxembourg)^35^.

### Injection of adenovirus and *in vivo* imaging

Ten-week-old male C57BL/6J mice were anesthetized with isoflurane before the injection through the penis vein with a 150 μl final volume of sterile physiological serum containing TRE-Luc (5 × 10^8^ pfu/mouse) and RSV-β-gal (10^8^ pfu/mouse) adenoviruses. Mice were analyzed 4 days after adenovirus delivery. For *in vivo* imaging, mice infected with indicated adenoviruses were anesthetized and injected (i.p.) with a dose of 30 mg/kg sterile firefly D-luciferin solution (Biosynth AG, Staad, Switzerland). After 3 min, mice were imaged on the Biospace Photon Imager (Biospace, Nesles-la-Vallée, France), and bioluminescence was analyzed with Biospace software. Luciferase activity was normalized to β-galactosidase activity used as an internal control for adenoviral infection.

### β-galactosidase determination

β-galactosidase assays for normalization of TRE-Luciferase activity were performed using hepatocyte or liver lysates, 2X buffer (1.33 mg/ml 2-nitrophenyl β-D-galactopyranoside, 100 mM 2-mercaptoethanol, 2 mM magnesium chloride, 200 mM sodium phosphate, pH 7.5) (Sigma, St. Louis MO) in each well of a clear 96-well plate. After 30 min of incubation at 37 °C, absorbance at 405 nm was determined with a Biorad Lumimark Plus plate reader (Marnes-la-Coquette, France). Lysate samples were assayed in triplicate. Lysates from non-transfected cells were used as controls for background activity. β-galactosidase activity was expressed as units/mg of protein.

### Analytical procedures

Serum insulin concentrations were determined using an insulin ELISA Assay Kit (Crystal Chem, INC, IL, USA) and a mouse insulin standard. Blood glucose was determined using one-touch AccuCheck® glucometer (Roche-Diagnostics, Meylan, France).

### Primary culture of hepatocytes and thransfection

Hepatocytes were isolated from livers of 8- to 10-week-old male C57BL/6 J mice by an *in situ* collagenase method as described previously^57^. Primary hepatocytes were cultured in M199 containing 5 (G5) or 25 mM glucose (G25) in the absence or the presence of 100 nM insulin for 24 h, an insulin concentration and time incubation time defined to provide maximal induction for the following experiments (Supplementary Fig. 5). For SCD1 overexpression/silencing experiments or Porcupine invalidation, hepatocytes were infected during 24 h with 3 pfu/cell of overexpressing-adenoviruses (GFP or SCD1), and/or sh-adenoviruses (SCD1i, PORCNi or unspecific inhibition USi,) and then cultured for additional 24 h. For PORCN pharmacological inhibition, primary hepatocytes were treated or not during 24 h with the IWP1 (inhibitor of Wnt process, 1 μg/ml) or DMSO. For palmitoleate/palmitate experiments, cells were incubated with M199 containing 200 μM albumin-bound palmitoleate (C16:1n7; Sigma-Aldrich, Saint-Quentin-Fallavier, France) or 250–500 μM albumin-bound palmitate (C16:0; Sigma-Aldrich) at a fatty acid/albumin ratio of 4:1 with fatty acid–free bovine serum albumin (Sigma-Aldrich). For insulin signaling inhibition, hepatocytes were treated with either 10 μM of Akti (Akti VIII, InVivoGen), 1 μM of wortmannin or 1 μM of rapamycin (Sigma-Aldrich) or DMSO as control, prior to incubation for 24 h in G25 containing 100 nM insulin. For luciferase reporter assays, primary hepatocytes were infected with the TRE-Luc reporter gene adenovirus and β-galactosidase reporter (RSV-β-gal) as an internal control as described previously^58^. Luciferase activity was measured on cell lysates 48 h after reporter infection and values were normalized for infection efficiency using β-galactosidase activity.

### Western blot analysis

Cultured cells or mouse livers were solubilized as described previously^21^. The protein extracts were subjected to SDS-PAGE electrophoresis and immunoblotted with the following antibodies: anti-pAkt (pS473, Cell Signaling #4060, Boston, MA, USA), anti-Akt (Cell Signaling #9272), anti-β-actin (Sigma-Aldrich #A5316), anti-β-catenin (BD Transduction Laboratories #610153, San Jose, CA, USA), anti-active-β-catenin (Merck-Millipore #05-665, NJ, USA), anti-Glutamine Synthetase (BD Transduction Laboratories #610517), anti-IRβ (Santa Cruz, SC-711, Heidelberg, Germany), anti-p-S6K (pThr 389, Cell Signaling #9234), anti-SCD1 (Cell Signaling #2794). The FAS antibody was a kind gift from Dr. I. Dugail (UMR-ICAN, Paris, France). The immunoreactive bands were revealed using the ECL detection kit (Pierce ECL Western Blotting substrate, Rockford, IL USA). Autoradiograms were quantified using an imageJ program (Chemi Genius2 scan, GeneSnap; Syngene, Cambridge, UK).

### Isolation of total RNA and analysis of mRNA expression by RT-qPCR

Total cellular RNAs from whole liver or from primary cultured hepatocytes were extracted using the SV Total RNA Isolation System (Promega, WI, USA). Total RNA (1 μg) was reverse-transcribed for 1 h at 42 °C in a reaction containing 50 mM Tris-HCl, 75 mM KCl, 3 mM MgCl_2_, 10 mM dithiotreitol, 250 mM random hexamers (Promega), 250 ng of oligo(dT) (Promega), 2 mM of each dNTPs, and 100 units of superscript II reverse transcriptase (Invitrogen, CA, USA). mRNA levels were measured by quantitative PCR using a Roche Light Cycler and the relative quantification for a given gene was corrected to the Cyclophilin mRNA values. Primer sequences are available on Supplementary Table 1.

### Equipment and settings

Blots from Figs. 1, 3 and Supplementary Figs. 2–4 were obtained by autoradiography using GE Healthcare Amersham HyperfilmTM ECL. Western blots were scanned using Epson Perfection 1670 Scanner. *In vivo* imaging (Figs. 1a and 2e) was performed using Biospace Photon Imager and analyzed with Biospace software (Biospace, Nesles-la-Vallée, France).

### Statistical analysis

Results are reported as means +/− SEM. Statistical analyses were performed with Prism software (GraphPad) using Mann-Whitney test or One-way ANOVA. Differences were considered statistically significant at p < 0.05.

## Supplementary information

Supplementary information.
