# Supplementary material for: Insulin activates hepatic Wnt/β-catenin signaling through stearoyl-CoA desaturase 1 and Porcupine
Source: Sci Rep. 2020 Mar 20;10:5186. doi: 10.1038/s41598-020-61869-4 (PMC7083857; doi:10.1038/s41598-020-61869-4)

Insulin activates hepatic Wnt/β-catenin signaling through stearoyl-CoA desaturase 1 and Porcupine

R. Cabrae, C. Dubuquoy, M. Cauzac, L. Morzyglod, S. Guilmeau, B. Noblet, C. Postic, B. Feve, A.-F. Burnol and M. Moldes

Supplementary table 1: Primer sequences

Name	Sequence primers
Lect2 sense	TAGCAGGACCATGGGCTAAC
Lect2 antisense	GCCCACTATCTTCCCAGTGA
GS sense	CAGGGACATCGTGGAGGCTC
GS antisense	TTGGAATTCCCACTGGGCAG
β-catenin sense	CTCTGAGCCCTAGTCATTGC
β-catenin antisense	TGCTGACTATCCAGTTGATGG
FAS sense	TTCCAAGACGAAAATGATGC
FAS antisense	AATTGTGGGATCAGGAGAGC
SCD1 sense	CCGGAGACCCTTAGATCGA
SCD1 antisense	TAGCCTGTAAAAGATTTCTGCAAA
Cyclophilin Sense	ATGGCACTGGTGGCAAGTCC
Cyclophilin antisense	TTGCCATTCCTGGACCCAAA

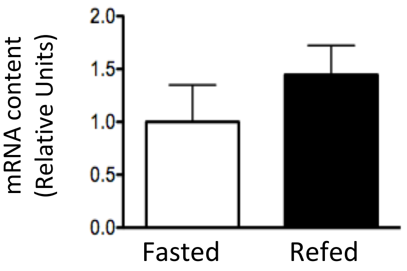

Supplementary Fig. 1: Hepatic β-catenin expression in fasted and refed mice
RT-qPCR analysis of β-catenin (*Ctnnb1*) gene expression in liver from fasted and refed mice. Results are the mean ± SEM (n=5/group).

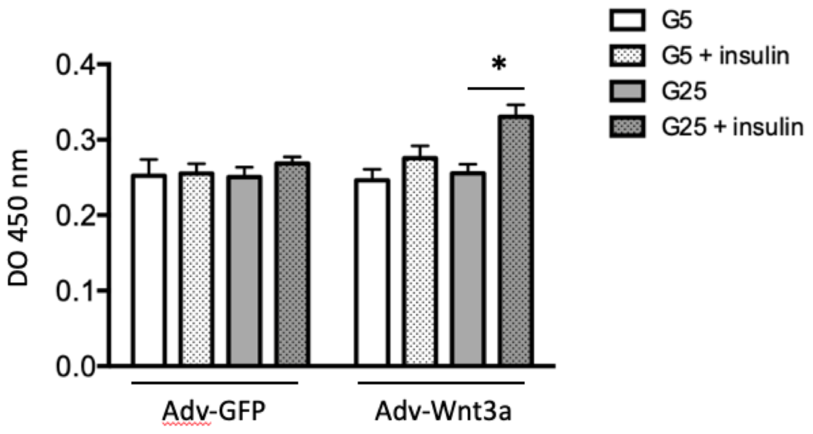

Supplementary Fig. 2: : Viability of primary hepatocytes is not altered in the absence of insulin.

Isolated primary hepatocytes were infected during 24 hours with an adenovirus overexpressing the ligand Wnt3a or the control GFP (1 pfu/cell). Cells were then treated with 5 mM or 25 mM glucose in the presence or the absence of 100 nM insulin during 24 hours. A MTT assay was performed. Results are the mean ± SEM (n=3 experiments, performed in triplicate). * $p<0.05$ G25+insulin *vs* G25.

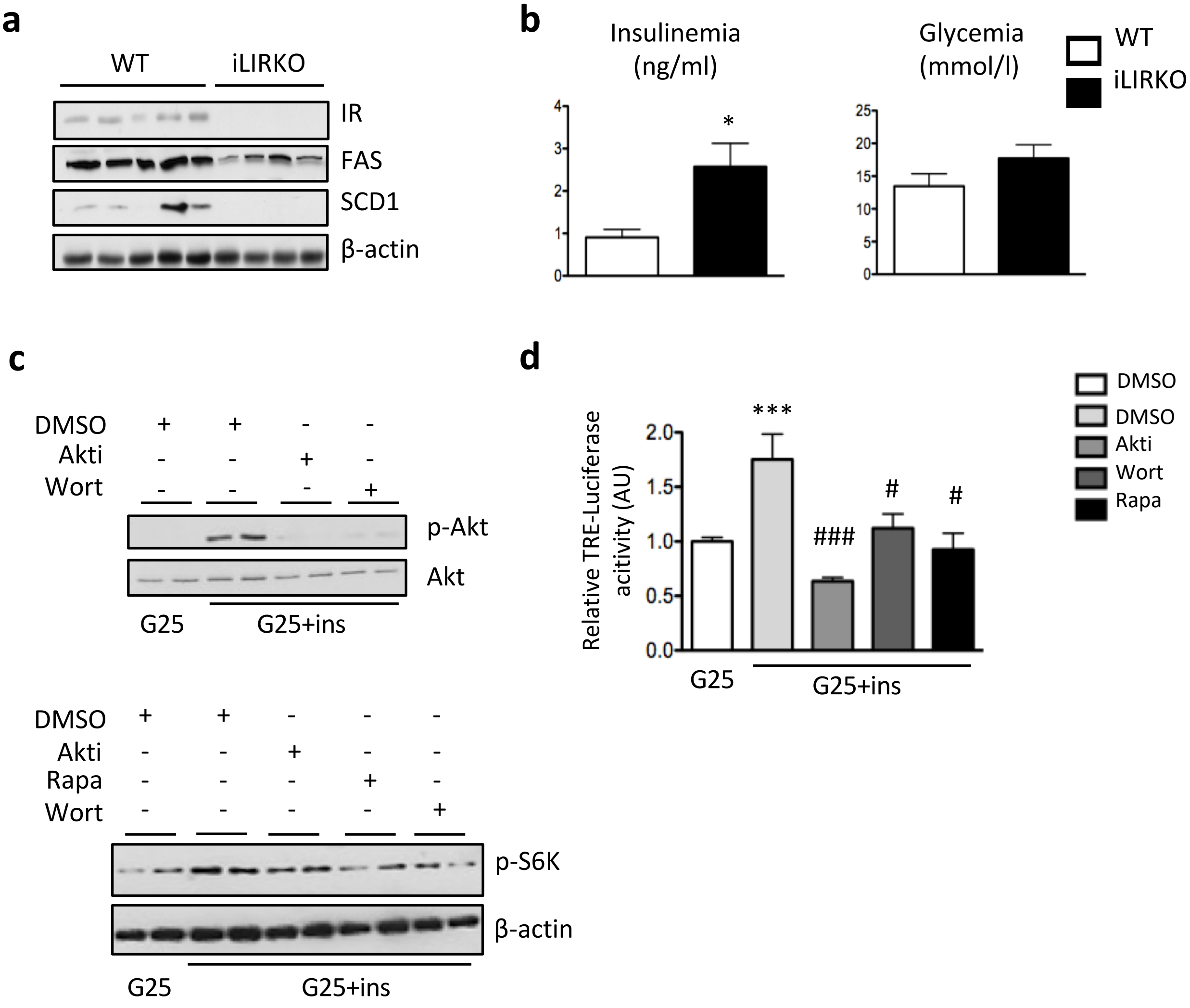

Supplementary Fig. 3: Hepatic Wnt pathway activity requires a functional insulin signaling
(**a-b**) iLIRKO and littermate control mice were sacrificed in the refed state. (A) Western blot analysis of protein content in liver lysates. β-Actin antibody was used as a loading control. (B) Insulinemia and glycemia of control and iLIRKO mice. Results are the means ± SEM (n=9-10/group). * $p<0.05$, iLIRKO *vs* control mice. (C-D) Mouse primary hepatocytes infected with the adenoviruses TRE-Luc and RSV-β-gal, were cultured in 25 mM glucose in the presence or in the absence of insulin and of insulin signaling inhibitors (Akti, wormannin, rapamycin) or control DMSO. (**c**) A representative Western blot analysis is shown for the effect of the inhibitors on insulin signaling. (**d**) Quantification of relative TRE-Luciferase activity was performed. Results are expressed as percent of the ratio firefly luciferase/β-galactosidase and are the mean ± SEM (n=3). *** $p<0.001$ *vs* G25, and # $p<0.05$; ### $p<0.001$ *vs* G25+ins+DMSO.

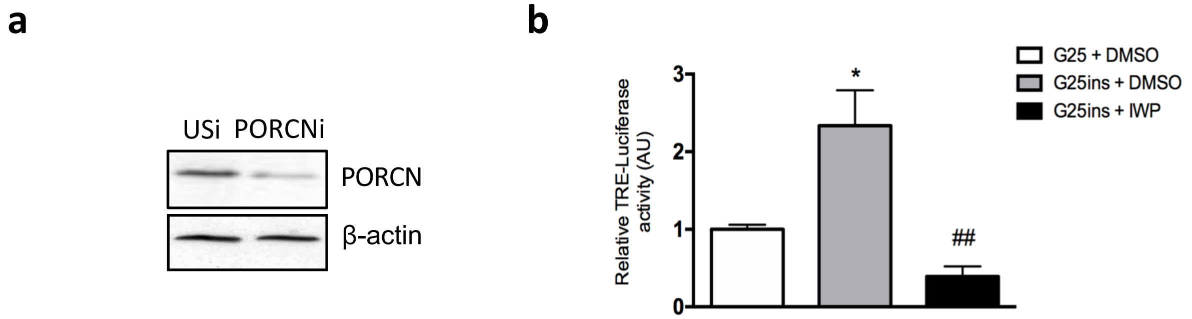

Supplementary Fig. 4: Efficiency of PORCN inhibition in hepatocytes

(**a**) Western blot analysis of PORCN protein content from primary mouse hepatocytes infected with PORCNi or USi adenoviruses and incubated in 25 mM glucose and 100 nM insulin for 24h. β-Actin antibody was used as a loading control. (**b**) Primary mouse hepatocytes were infected with Adv-TRE-Luc and Adv-RSV-β-gal and incubated with G25 with or without insulin (100 nM) during 24 hours in the presence of the absence of IWP1 (1µg/ml). Ratio of luciferase / β-galactosidase activity was determined. Results are the mean ± SEM (n=3 experiments, performed in triplicate). * $p<0.05$ *vs* without insulin; ##<0.01 *vs* without IWP1 treatment.

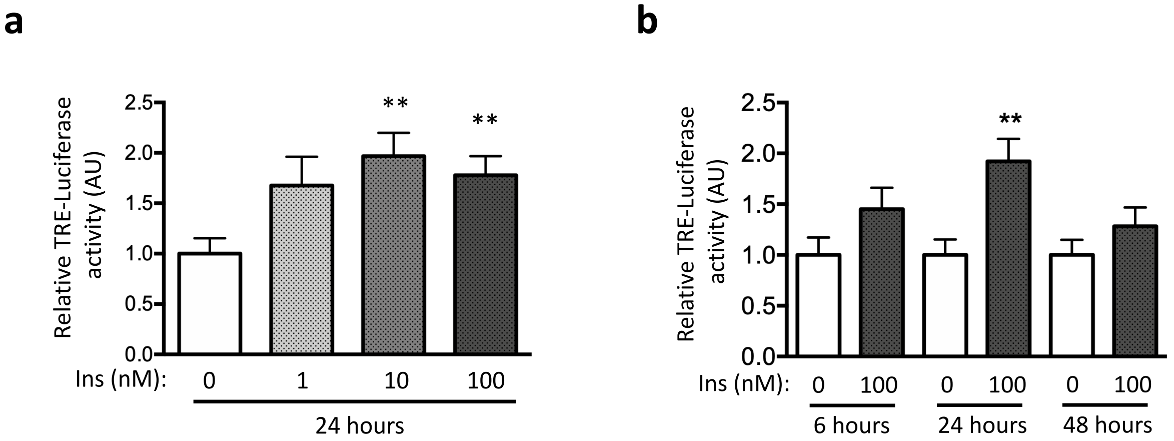

Supplementary Fig. 5: Time- and dose-dependent activity of TRE-Luc in primary cultured hepatocytes.

Isolated primary hepatocytes were incubated during 24 hours with 1, 10 or 100 nM of insulin (**a**) or with 100 nM insulin during 6, 24 and 48 hours (**b**). Prior to incubation, cells were infected with adenoviruses overexpressing TRE-Luc and β-galactosidase. The ratio of luciferase/β-galactosidase activity was determined. Results are the mean ± SEM (n=3 experiments, performed in triplicate). ** $p<0.01$ *vs* without insulin.

Uncut blots:

Figure 1c : Fasted/refed mice

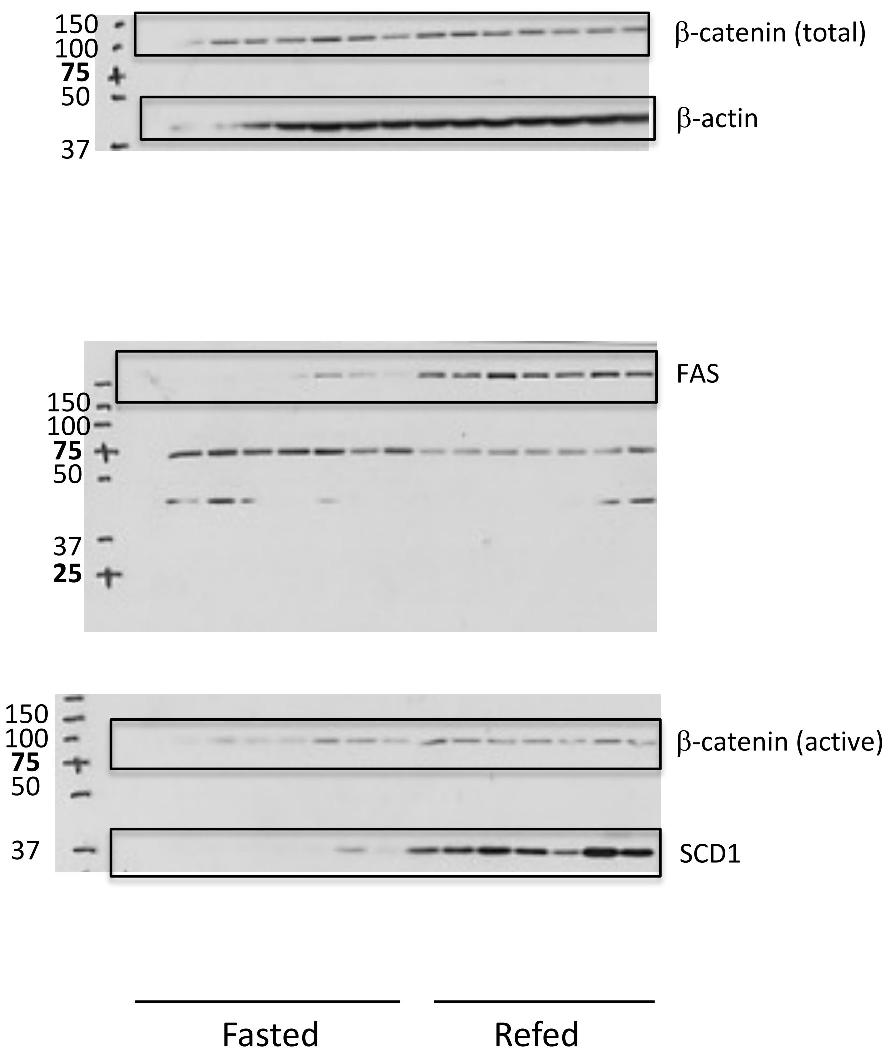

Uncut blots:

Figure 3c : SCD1 western blot

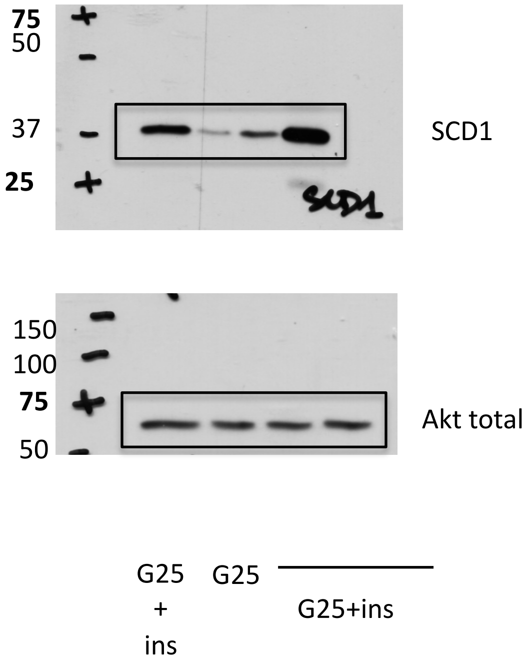

Uncut blots:

Supplementary figure 2a : iLIRKO mice

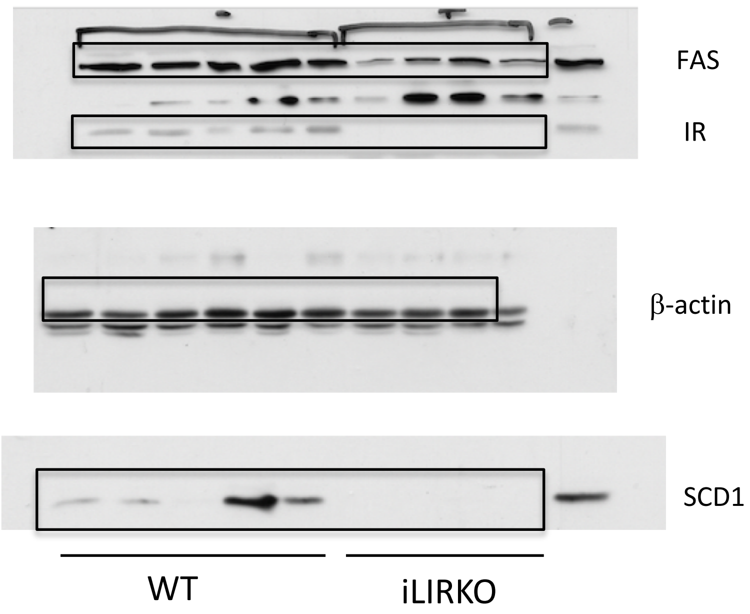

Uncut blots:

Supplementary figure 2c : Insulin Inhibitors

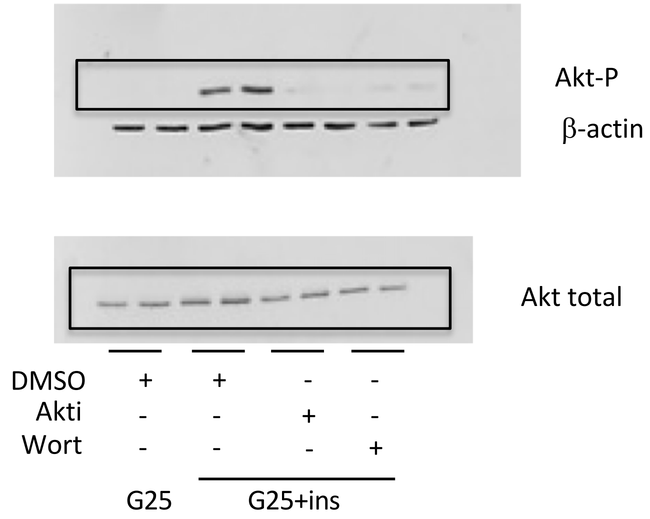

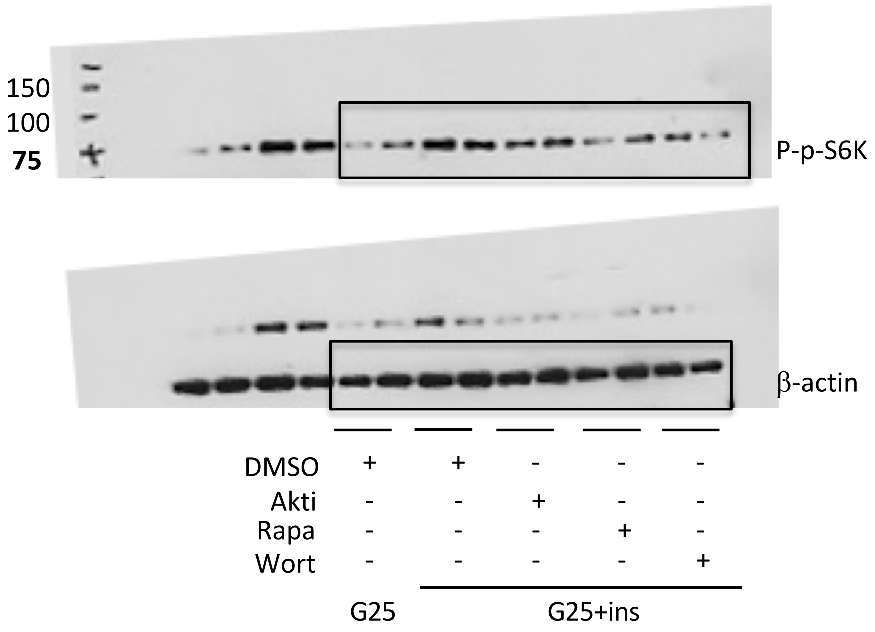

Supplement: Supplementary file 1 — Supplementary information. [file 41598_2020_61869_MOESM1_ESM.pdf]
